# Assessment of 19 Operation Room and Sterile Processing Units in Puerto Rico, 2023: Preliminary Findings using a new ICAR Tool

**DOI:** 10.1017/ash.2024.254

**Published:** 2024-09-16

**Authors:** David Nachi, Norma I. Diaz-Paris, Jonell Gonzalez-Pagan, Miguel A. Jorge-Coreano, Tanialy Rivera-Santiago, Angelica Garcia-Segui, Sylvianette Luna-Anavitate, Melissa Marzan-Rodriguez

**Affiliations:** Puerto Rico Department of Health; University of Puerto Rico Comprehensive Cancer Center

## Abstract

**Background:** Infection prevention and control assessments in healthcare settings serve as a primary resource for obtaining data and providing recommendations based on safety, compliance, and quality assurance guidelines. In Puerto Rico (PR), surgical site infections are underreported in the Epi Info platform used by the Puerto Rico Department of Health (PRDOH), mainly due to the complexity of their identification. By focusing on evaluating Operating Rooms/Sterile Processing and Distribution (OR/SPD) units in acute care facilities (ACFs), our goal is to generate new data within the Healthcare-Associated Infection/Antibiotic Resistance (HAI/AR) Program, specifically related to patient management throughout preoperative, intraoperative, and postoperative phases, as well as reprocessing practices. **Methods:** Nineteen evaluations of ACFs' OR/SPDs were conducted from May through December 2023. Direct observations, file reviews, and personnel assessments were performed using an infection control assessment and response (ICAR) tool developed collaboratively by a team from an acute facility in PR and the HAI/AR Program staff. This ICAR Tool was customized based on guidelines from the certified Board for Sterile Processing and Distribution (CBSPD), the Association of periOperative Registered Nurses (AORN), and the Association for the Advancement of Medical Instrumentation (AAMI), among other regulatory agencies. The Division of Health Quality Promotion (DHQP) reviewed and approved the tool for use in these evaluations. **Results:** Key findings indicate that 32% of Sterile Processing Department (SPD) units restrict access to dedicated personnel with available manufacturer’s instructions, yet only 36% of SPD personnel are certified in CBSPD and packaging practices. Only 10% of facilities had a water treatment system for sterilization and Immediate Use Steam Sterilization (IUSS) policies. Notably, 84% of endoscopy areas require additional equipment for cultivating endoscopes, and no facility possessed a borescope for visually inspecting endoscope lumens. Tray inspection occurred in 21%, and only 31% of staff knew the Spaulding Classification and Class V Indicators. **Conclusion:** These data underscore the necessity of evaluating OR/SPD units in ACFs to provide updated recommendations and mitigate the incidence of surgical site infections (SSI). They offer insight into the structural and functional status of OR/SPD units in Puerto Rico, aligning reporting with OR/SPD practices to enhance patient care and minimize infection risks.

